# Prospective association between adherence to UK dietary guidelines in school-age children and cardiometabolic risk markers in adolescence/early adulthood in the Avon Longitudinal Study of Parents and Children (ALSPAC) cohort

**DOI:** 10.1017/S0007114523000685

**Published:** 2023-11-28

**Authors:** Genevieve Buckland, Caroline M. Taylor, Pauline M. Emmett, Kate Northstone

**Affiliations:** 1 Centre for Academic Child Health, Bristol Medical School, University of Bristol, Bristol, UK; 2 Department of Population Health Sciences, Bristol Medical School, University of Bristol, Bristol, UK

**Keywords:** Eatwell Guide, UK dietary recommendations, Cardiometabolic risk score, Children and adolescents, Avon Longitudinal Study of Parents and Children (ALSPAC), Prospective Cohort Study

## Abstract

Research into how alignment to UK dietary guidelines during childhood affects cardiometabolic health is limited. The association between adherence to UK dietary guidelines during childhood and overall cardiometabolic risk (CMR) in adolescence/early adulthood was explored using data from the Avon Longitudinal Study of Parents and Children (ALSPAC). ALSPAC children with diet diaries completed at 7, 10 and 13 years of age, and data on CMR markers at 17 years (*n* 1940) and 24 years (*n* 1957) were included. A children’s Eatwell Guide (C-EWG) score was created by comparing dietary intakes at each age to UK dietary guidelines for nine foods/nutrients. Cardiometabolic health at 17 and 24 years was assessed using a composite CMR score. Multivariable linear regression models examined associations between C-EWG scores at 7, 10 and 13 years and the CMR score at 17 and 24 years, adjusting for confounders. C-EWG scores were generally low. However, a higher score (adherence to more dietary guidelines) at 7 years old was associated with a lower CMR score at 17 and 24 years: *β* −0·13 (95 % CI −0·25, –0·01) and *β* −0·25 (95 % CI −0·38, –0·13) for a 1-point increase in C-EWG score, respectively. A higher C-EWG score at 10 years was also associated with a lower CMR z-score at 24 years. No clear associations were evident at other ages. Greater adherence to UK dietary guidelines during mid-childhood was associated with a better overall cardiometabolic profile, suggesting that encouraging children to eat in this way has long-term benefits to health.

Unhealthy dietary habits are a major modifiable risk factor contributing to the high burden of disability-adjusted life years and premature mortality from non-communicable chronic diseases^(1)^. The Global Burden of Disease 2019 report estimated that 7·94 million annual deaths and 188 annual disability-adjusted life years were attributed to fifteen dietary risk factors: underconsumption of fruits, vegetables, legumes, whole grains, nuts and seeds, milk, fibre, Ca, *n*-3 fatty acids from seafood and PUFA and overconsumption of red and processed meat, sugar-sweetened beverages, trans-fatty acids and Na^(2)^.

National and international dietary guidelines aim to improve the well-being and long-term health of populations, including reducing the prevalence of CVD, type II diabetes (T2D) and their risk factors^(3,4)^. For instance, UK dietary guidelines have been developed by the UK Health Security Agency (UKHSA), previously known as Public Health England, in partnership with the Scientific Advisory Committee on Nutrition after reviewing the evidence-base on diet and health^(3)^. Their current recommendations for adults^(3,5,6)^ align closely to international dietary guidelines^(4)^ and are visually represented within the UK’s Eatwell Guide (EWG)^(5)^.

Epidemiological studies have been used to assess and quantify the population health benefits from adhering to such dietary recommendations^(4,7–13)^. The UK Biobank study reported that adults adhering to three or four WHO dietary recommendations, for saturated fats, free sugars, fibre, and fruit and vegetables, had a 21 % reduced risk of all-cause mortality and a 22 % reduced risk of fatal CVD^(4)^. Meeting more dietary recommendations was also associated with better cardiometabolic health parameters^(4)^. Another study in UK adults found that greater alignment to eight core UK dietary recommendations, assessed using a dietary reference value index, was associated with a decrease in several cardiometabolic risk (CMR) factors, including waist circumference, BMI, total cholesterol and blood glucose^(7)^. An EWG recommendations score was recently developed to explore the association between meeting nine core UK dietary recommendations and all-cause mortality in an analysis of multiple UK observational studies: compared with a very poor EWG adherence score, having an intermediate-to-high EWG adherence score was associated with a 7 % reduced risk of mortality^(11)^.

In the UK, 24 % of all deaths are caused by heart and circulatory diseases, and 28 % of these are premature (under 75 years)^(14)^. Cardiometabolic diseases (CVD and T2D) have been linked to a life-course accumulative exposure to unhealthy diets^(15,16)^. However, there is limited research in paediatric populations assessing whether adherence to UK government dietary recommendations is related to better cardiometabolic health. Childhood is a critical period when dietary habits are becoming established and begin to influence the emergence and trajectories of CMR factors such as excess weight gain, elevated blood pressure, and raised lipid and glucose levels^(17,18)^. These CMR markers provide useful intermediate pre-clinical measures of cardiometabolic health in younger populations before overt cardiometabolic diseases are evident^(19)^. Although there is increasing evidence showing that healthy dietary patterns during childhood and adolescence are linked to better measures of individual CMR markers^(20–22)^, to our knowledge no study has specifically studied if adherence to UK dietary guidelines throughout childhood affects overall cardiometabolic health. This is also relevant because previous research from our group showed that dietary intakes of school-age children in a population-based UK birth cohort (Avon Longitudinal Study of Parents and Children (ALSPAC)) were mostly suboptimal compared with UK dietary recommendations^(23)^.

This study assessed the prospective association between adherence to UK national dietary guidelines in children aged 7–13 years, measured using a children’s Eatwell Guide (C-EWG) score, and cardiometabolic health in late adolescence/early adulthood, measured using a composite CMR score.

## Methods

### Cohort description

**Fig. 1.**
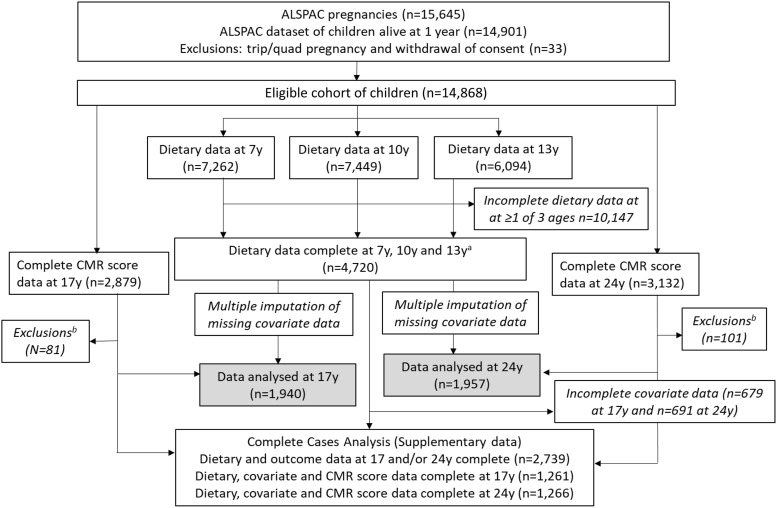
Study flow diagram for participant data from the Avon Longitudinal Study of Parents and Children (ALSPAC). The present study uses data from participants with complete dietary data at 7, 10 and 13 years and complete data on the cardiometabolic parameters to derive the cardiometabolic risk (CMR) score at 17 and 24 years and uses multiple imputation for missing data in covariates. ^a^Complete dietary data refer to at least one diet diary recorded for a child at all three ages (7, 10 and 13 years). Three complete days of diet diary data were available for 86·5, 83·6 and 78·4 % of children at 7, 10 and 13 years, respectively. ^b^Exclusions were participants with diagnosed diabetes, on insulin treatment or fasting glucose level ≥ 7 mmol/l and participants with extreme outlying data, defined as more than 4 sd from the mean, on any of the six CMR score components.

This research is based on ALSPAC, an ongoing British birth cohort study established in the 1990s to investigate the determinants of health and disease across the life course^(24)^. Full details of the study are also available on the ALSPAC website (www.alspac.bris.ac.UK) and have been previously reported^(25–27)^. In brief, 14 541 eligible pregnant women from the South-West of England were initially enrolled into the study in 1991–1992, resulting in 13 988 children alive at 1 year. Three subsequent recruitment phases^(27)^ resulted in an additional 913 children being enrolled and increased the cohort sample to 15 454 pregnancies and 14 901 children alive at 1 year (*n* 14 868 eligible children after excluding participants who withdrew consent and triplet and quadruplet pregnancies for reasons of confidentiality). The current study is based on this initial sample of 14 868 ALSPAC children, with subsequent exclusions of children with missing outcome and exposure data collected during follow-up (Fig. 1). At birth, ALSPAC children were relatively representative of the population in the area^(25)^. Extensive data have been periodically collected from the parents and their children during over 30 years of follow-up, primarily using questionnaires, medical records and face-to-face visits. The Research Electronic Data Capture (REDCap) electronic data capture tool hosted at the University of Bristol is used to collect and manage the data^(28)^. REDCap is a secure, web-based software platform designed to support data capture for research studies. The study website contains details of all the data that are available through a fully searchable data dictionary and variable search tool (http://www.bristol.ac.UK/alspac/researchers/our-data/).

### Ethical approval and informed consent

Ethical approval for the study was obtained from the ALSPAC Ethics and Law Committee and the Local Research Ethics Committee (http://www.bristol.ac.UK/alspac/researchers/research-ethics/), and the study conformed to the guidelines within the Declaration of Helsinki. Consent for biological samples was collected in accordance with the Human Tissue Act (2004). Written or verbal informed consent for the use of data collected via questionnaires and clinics was obtained from participants following the recommendations of the ALSPAC Ethics and Law Committee at the time. All children were invited to give consent at every follow-up, where appropriate. Full details of the ALSPAC consent procedures are available on the study website (http://www.bristol.ac.UK/alspac/researchers/research-ethics/).

### Dietary assessment

Full details of the ALSPAC dietary assessment methodology have been previously published^(29)^. In summary, dietary data were collected at 7, 10 and 13 years using 3-d diet diaries, for which participants recorded all food and drink consumed by the child over two weekdays and one weekend day. The diet diaries were completed by the caregivers when the children were 7 years old and by the children with support from the caregiver when the children were 10 and 13 years old. The children then attended a research clinic (mean age at attendance: 7·5 (sd 0·3), 10·6 (sd 0·2) and 13·8 (sd 0·2)) years) where a nutritionist checked the diaries for completeness or discrepancies and clarified portion sizes. The diet diary data were coded and linked to food composition tables using Diet In Data Out. Nutrient intakes were calculated using McCance and Widdowson’s British food composition tables^(30)^. Plausibility of dietary reporting was calculated using an individualised method based on the ratio of energy intake (EI) to estimated energy requirement (EER) and its 95 % CI^(31,32)^. Individualised EER are calculated from estimates of total energy expenditure and energy required for growth, and use body weight equations from Torun^(33)^. The EI/EER ratio and overall CV_t_ of EI/EER were used to establish the accuracy of reported EI; if EI/EER is one or 100 %, then reported EI was considered valid. A 95 % CI for EI:EER was calculated to take into account the variation in the accuracy of estimating EI and EER^(31)^. Individuals were classified as under-reporters if EI:EER was below the 95 % CI (< 78·45 %) and as over-reporters if EI:EER was above the 95 % CI (> 121·55 %). Data from dietary diaries were available for 7262 children at 7 years, for 7449 at 10 years and for 6094 at 13 years and 4720 children had dietary data at all three ages (Fig. 1).

### Children’s Eatwell Guide score

A C-EWG score was based on previously published methods^(11,23)^. It was previously used in the same cohort of ALSPAC children to evaluate the socio-economic predictors of having low C-EWG scores at 7, 10 and 13 years of age^(23)^. The score represents the number of core UK dietary recommendations met by the children at 7, 10 and 13 years old. At each age, the children’s dietary intakes were compared with current UK dietary recommendations outlined in the UKHSA 2016 Report and EWG^(5,6)^, and the nutrient-specific reports this is based on^(5,34–38)^. Nine foods/nutrients were assessed: total fat, saturated fat, free sugars, fibre, salt, fruit and vegetables, non-oily fish, oily fish and red/processed meat^(11)^. The UK recommended minimum intakes or constraints for each of these foods and nutrients at the corresponding ages of the participants (7, 10 and 13 years) are detailed in Table 1. Recommendations for fruit and vegetables, fish and red/processed meat are all specified in terms of number of portions, with portion sizes defined as grams for adults^(5)^. We adjusted these adult portion sizes to child-appropriate portion sizes for 7-, 10- and 13-year-old children, based on a previously published method^(39)^ which takes into account dietary reference values for energy at each age to calculate portion sizes which were proportionally less at younger ages^(37)^. Each child’s intake of the nine foods/nutrients was categorised into meeting (1 point) or not meeting (0 points) the dietary guidelines at each age. The C-EWG score is the sum of the points obtained from each of the nine foods/nutrients and therefore ranges from 0 to 9 (none to all recommendations met).

**Table 1.** UK dietary recommendations for key nutrients and foods within the Eatwell Guide, including age-adjusted portion sizes calculated for the ALSPAC children at 7, 10 and 13 years old

UK Dietary Recommendations	7 years	10 years	13 years
*Recommended intakes of nutrients*			
Total Fat^a^	≤35% total energy	≤35% total energy	≤35% total energy
Saturated fat^a^	≤11% food energy^b^	≤11% food energy^b^	≤11% food energy^b^
Free sugars^c^	≤5% total energy	≤5% total energy	≤5% total energy
Salt^d^	≤5g/day	≤5g/day	≤6g/day
Fibre (AOAC)^c^	≥20g/day	≥20g/day	≥25g/day
*Recommended intakes of foods* ^*e*^			
Fruit and Vegetables (f&v)^f^	≥5 portions/day	≥5 portions/day	≥5 portions/day
	*1 portion f&v* ^*g*^ * = 50g*	*1 portion f&v* ^*g*^ * = 65g*	*1 portion f&v* ^*g*^ * = 75g*
	*1 portion dried fruit = 20g*	*1 portion dried fruit = 25g*	*1 portion dried fruit = 30g*
	*1 portion of beans/ pulses = 55g* ^*h*^	*1 portion of beans/ pulses = 65g* ^*h*^	*1 portion of beans/ pulses = 75g* ^*h*^
	*1 portion of fruit/vegetable juice or smoothie = 100ml* ^*h*^	*1 portion of fruit/vegetable juice or smoothie = 125ml* ^*h*^	*1 portion of fruit/vegetable juice or smoothie = 145ml* ^*h*^
Fish total (includes 1 portion/week of oily fish)^f^	2 portion/week (190g/week)	2 portion/week (230g/week)	2 portion/week (270g/week)
Oily fish^f^	1 portion/week (95g/week)	1 portion/week (115g/week)	1 portion/week (135g/week)
Red/processed meat^f^	≤45g/day	≤55g/day	≤65g/day

^a^SACN report on saturated fats and health (2019) [29]. ^b^COMA Dietary reference value report 1991 states recommendation of ≤11% of energy refers to food energy (excluding alcohol) [29,30]. ^c^SACN recommendations on carbohydrates, including sugars and fibre (2015) [27]. ^d^SACN Report on Salt and Health (2003) [28]. ^e^Age appropriate portion sizes defined using previously published methods [32]. ^f^Public Health England - The Eatwell Guide: How does it differ to the eatwell plate and why (2016) [5]. ^g^Fruit and vegetables portions include fresh, canned or frozen fruit and vegetables (excluding potatoes) [5]. ^h^These food groups can only count towards 1 of the 5 portions/day of fruit and vegetables [5].

### Cardiometabolic risk factors

CMR factors were assessed when the participants attended study clinics at a mean age of 17·7 (sd 0·3) years and 24·5 (sd 0·8) years. Study nurses and clinic staff took anthropometric and blood pressure measurements and blood samples using standard procedures, detailed in full elsewhere^(40)^. Blood pressure was measured in a seated position and resting state, using validated electronic monitoring devises (Omron). Systolic blood pressure (SBP) and diastolic blood pressure (DBP) were measured twice on the right arm, using the appropriate cuff size for the upper arm circumference, and the mean of each was recorded. Mean arterial blood pressure (MAP) was calculated using the formula 1/3(SBP)+2/3(DBP)^(41)^. Blood samples were taken after fasting overnight for at least 6–8 h before the clinic visit. The samples were immediately centrifuged and frozen at −80°C and were assayed 3–9 months later with no previous freeze–thawing cycles. Plasma lipids were analysed using the standard Lipid Research Clinics Protocol with enzymatic reagents for lipid determination. Insulin and glucose measurements were used to calculate the homeostatic model assessment of insulin resistance (HOMA-IR) using the following standard formula: (fasting plasma glucose (mg/dl) × fasting plasma insulin (mU/l))/405^(42)^. Fat mass (kg) was measured at 17 and 24 years using a Lunar Prodigy Dual Emission X-ray Absorptiometry (DXA) scanner (GE Medical Systems) and fat mass index (FMI) was calculated as fat mass (kg)/height (m)^2^. Weight, height and waist circumference were also measured at each clinical visit. Weight was measured using the Tanita Body Fat Analyser weighing scale (Tanita), height using a Harpenden stadiometer (Holtain Ltd) and waist circumference using a Seca 201 body tension tape. Height and waist circumference were measured to the nearest millimetre, while weight was measured to the nearest 0·1 kg. BMI was calculated as weight(kg)/height(m)^2^.

### Cardiometabolic risk score

A composite CMR score^(19)^ was calculated for each participant at 17 and 24 years, to provide a measure of overall cardiometabolic health at each age. Six cardiometabolic markers were included in the CMR score^(40)^: FMI, LDL-cholesterol, HDL-cholesterol, TAG, MAP and HOMA-IR. Data on all individual CMR markers were available for 2879 participants at 17 years and 3132 participants at 24 years. Due to potential problems of using HOMA-IR to assess insulin sensitivity in participants with diabetes^(43)^, we excluded participants with diagnosed diabetes (*n* 17) or on insulin treatment (*n* 15), as well as participants with fasting glucose concentration ≥ 7 mmol/l but who had not reported diabetes (*n* 4 at 17 years and *n* 14 at 24 years). Participants were also excluded if they had extreme/implausible values (≥ 4 sd from the mean) on any of the six CMR markers (*n* 42 at 17 years and *n* 52 at 24 years). Therefore, CMR scores were calculated for participants who had complete dietary data and complete outcome data at 17 years (*n* 1940) and 24 years (*n* 1957). Sex-specific z-scores were calculated for each CMR marker and HDL-cholesterol was multiplied by –1, to align the direction of values for increased risk with the other components. Each participant’s z-scores for the six CMR markers were totalled to give an overall CMR score at 17 years and at 24 years^(19)^. Higher CMR scores indicated worse cardiometabolic health relative to participants with lower CMR scores.

### Covariates

Participant covariate data were collected primarily through self-completion questionnaires, hospital and medical records and periodic face-to-face clinical assessment visits, described in full previously^(26)^. Maternal pre-pregnancy anthropometric data were collected by self-completion postal questionnaires during pregnancy^(24)^. Maternal educational attainment was recorded as the highest completed out of Certificate of Secondary Education, vocational training, O-level/General Certificate of Secondary Education (qualifications obtained at 16 years of age), A-levels (qualification obtained at 18 years), University degree or higher. Social class was derived using the 1991 Office of Population Censuses and Surveys occupation-based classification, based on the parents’ current or last job at 32 weeks of gestation. Standardised UK social class classifications were used which ranged from social class I to V (highest–lowest)^(44)^. Highest family social class was derived by combining maternal and paternal social class. Physical activity was assessed at 11 and 13 years of age using an Actigraph AM7164 2·2 accelerometer (Actigraph LLC), which was worn around the waist, at the right hip, for 7 consecutive days^(45)^. A valid day was defined as providing data for at least 10 h, and participants were only included in the analyses if they provided at least 3 valid days of recording. Moderate-to-vigorous physical activity was calculated using the mean minutes per day in which there were > 3600 accelerometer counts per minute^(45)^. Puberty timing was estimated using peak height velocity, previously calculated in ALSPAC^(46)^. Validity of dietary reporting was categorised into under-reporting, plausible reporting or over-reporting.

### Statistical analysis

The analysis was carried out on data from the children who had diet diaries collected at all three ages and CMR markers measured at 17 years and/or 24 years (*n* 2739). The baseline characteristics of these participants were described according to the C-EWG score (0, 1, 2 and ≥ 3 recommendations met) at the three ages, using proportions or means and standard deviations. Chi-squared tests and Kruskal–Wallis tests were used to assess differences between categorical and continuous variables, respectively. The study sample with complete dietary and CMR score data was compared with the excluded participants with incomplete dietary or outcome data using the aforementioned tests and also using Cohen’s d effect estimate for continuous variables.

Several factors were considered as potential confounders of the association between the C-EWG score and CMR score: child characteristics (sex, age at clinic session), dietary-related factors (number of diet diaries collected, plausibility of dietary reporting, total EI), perinatal/biological factors (birth weight, gestational age, puberty timing, maternal pre-pregnancy BMI), social factors (maternal age at delivery, maternal highest education attainment, highest family social class) and lifestyle factors (moderate-to-vigorous physical activity level of children at 11 and 13 years, smoking status and alcohol consumption of children at 17 and 24 years). The final fully adjusted regression models only included the set of covariates which were related to both the exposure and outcome (*P* < 0·10). The variance inflation factors for the covariates in all the different models were all ≤ 5, so the assumption of no multicollinearity was supported.

The association between the C-EWG score at 7, 10 and 13 years (exposure variables) and CMR z-score at 17 years and at 24 years (outcome variables) was calculated using unadjusted, minimally adjusted (sex and plausibility of dietary reporting) and fully adjusted multivariable linear regression models (additionally adjusted for mother’s highest education attainment, highest family social class, moderate-to-vigorous physical activity at 11 years and 13 years). The C-EWG score was evaluated as a categorical variable (0 – reference, 1, 2 and ≥ 3 points) and a continuous variable (per 1-point increase) based on previous methodology^(4)^. The CMR score was assessed as a continuous variable (per 1-unit increment), based on previous methodology^(40)^. There was no evidence of effect modification by sex (lrtest comparing models with and without an interaction term between the C-EWG score and sex resulted in all *P*-values > 0·10), so results are presented for both sexes together.

Multivariable linear regression models were also used to explore the association between the C-EWG score and each individual CMR marker within the score (FMI, HDL-cholesterol, LDL-cholesterol, TAG, MAP and HOMA-IR) and additional CMR markers (BMI, waist circumference, SPB, DBP, total cholesterol, insulin and glucose). All statistical analyses were performed using Stata version 15.1 (Stata Corporation).

### Missing data and multiple imputation

Multiple imputation was used to deal with missing data in covariates variables included in the regression models. Chained equations (ICE command) in Stata generated twenty stacked datasets which were used in the final regression analyses, with standard combination rules for multiple imputations^(47)^. The variables included in the imputation models were all of those included in the final regression models and additional auxiliary variables which also strongly predicted missingness in the covariates (Family Adversity Index^(48)^, maternal pre-pregnancy BMI, child’s BMI and total EI at each time of dietary data collection). Thus, the basic assumption underlying multiple imputation that data were ‘missing at random’^(47)^ was supported. Separate imputed datasets were created for the participants with complete dietary and CMR data at 17 years (*n* 1940) and at 24 years (*n* 1957). These imputed datasets were used for the multivariable regression analyses presented in the main article. The results from the complete-case analyses for CMR at 17 years (*n* 1261) and CMR at 24 years (*n* 1266) are presented in online Supplementary Tables 1 and 2. The distribution of covariates in the imputed and observed datasets was compared using chi-squared tests or Kruskal–Wallis tests (online Supplementary Table 3).

## Results

The final study sample included 2739 participants with C-EWG scores calculated at 7 and 10 and 13 years and CMR scores calculated at least once during follow-up; 1940 participants (50·1 % female) had CMR scores at 17 years and 1957 participants (56·8 % female) had CMR scores at 24 years (*n* 1158 had CMR scores available at both ages). The CMR score at 17 years had a mean of 0 (sd 3·6) and ranged from −7·7 to 16·7, and the CMR at 24 years had a mean of 0 (sd 3·8) and ranged from −9·4 to 17·8 (data not tabulated).

**Fig. 2.**
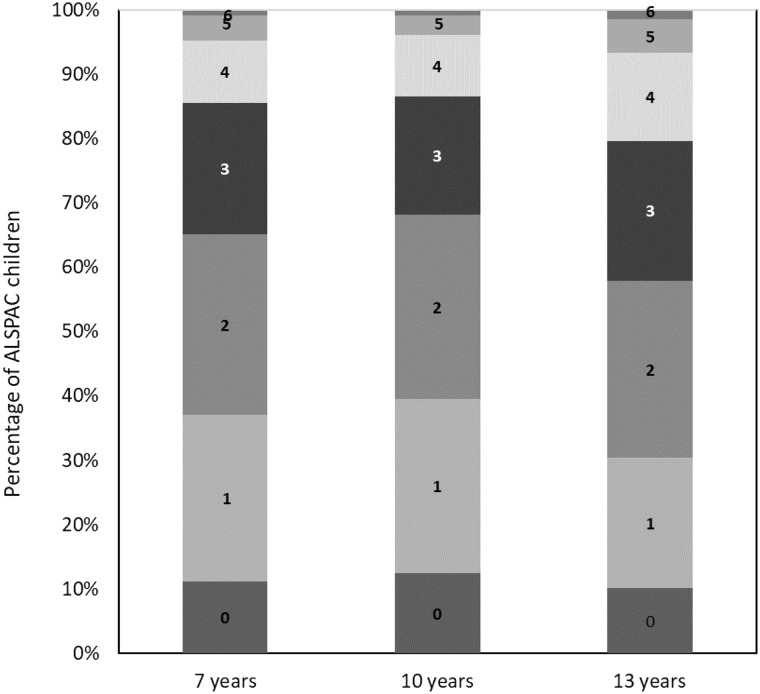
Percentage of Avon Longitudinal Study of Parents and Children (ALSPAC) children (*n* 2739) at 7, 10 and 13 years meeting different number of recommendations (ranging from 0 to 9) within the children’s Eatwell Guide (C-EWG).

The daily intake of the foods/nutrients included within the C-EWG score at 7, 10 and 13 years and percentage of the 2739 children adhering to the dietary recommendations for these foods/nutrients are detailed in online Supplementary Tables 4 and 5. In general, adherence to guidelines was very low (< 10 % of children) for free sugars, fish, saturated fat and fibre and was low-to-moderate (∼10 to 40 %) for meeting fruit and vegetables and salt recommendations. The mean C-EWG was 2·1 (sd 1·3) at 7 years, 2·0 (sd 1·3) at 10 years and 2·3 (sd 1·4) at 13 years and at all ages ranged from 0 points (no dietary recommendations met) to 7 points (7 out of 9 dietary recommendations met). Figure 2 illustrates the proportion of children meeting different number of EWG recommendations at each age; approximately 60 % of the cohort met less than three of the nine C-EWG recommendations.

The characteristics of the participants (*n* 2739) according to the C-EWG score are presented in Table 2. In general, children with higher C-EWG scores (meeting more dietary recommendations) compared with lower C-EWG scores were more likely to be female, had a lower BMI, consumed less dietary energy and had older mothers with a lower BMI, a higher education level and higher family social class. Participants excluded from the main analysis due to missing/incomplete dietary data and/or CMR outcome data were compared with those in the final study sample in online Supplementary Table 6. Overall, participants who were excluded were more likely to be male, have a higher BMI at 10 years, have younger mothers with a lower level of educational attainment and a lower family social class. Participants who were excluded generally had a worse cardiometabolic profile at 17 and 24 years. However, Cohen’s effect sizes, representing the standardised mean difference in these characteristics between the excluded and included participants, were generally low (d ≤ 0·2).

**Table 2.** Characteristics of the study sample with complete dietary and outcome data from at least one time-point, according to number of UK dietary recommendations met within the children’s Eatwell Guide (C-EWG) score at 7, 10 and 13 years

Characteristics of the ALSPAC children	C-EWG Score^a^					
	Total	0	1	2	≥3	P-value
**C-EWG score at 7 years, n**	**2,739**	**305**	**709**	**770**	**955**	
Age^b^, mean (sd) years	2,739	7·5 (0·2)	7·5 (0·3)	7·5 (0·3)	7·5 (0·3)	0·218^d^
BMI^c^, mean (sd) kg/m^2^	2,729	16·2 (1·8)	16·2 (2·0)	16·0 (1·9)	16·1 (1·9)	0·023^d^
Total Energy, mean (sd) MJ/day	2,739	7·7 (1·2)	7·5 (1·2)	7·1 (1·2)	7·0 (1·3)	<0·001^d^
Sex, % female	2,739	43·0	51·5	55·1	56·3	<0·001^e^
Validity of dietary reporting, % Under-reporters	2,729	4·6	6·4	10·9	13·0	<0·001^e^
Maternal age at delivery, % ≥30 years	2,632	41·8	47·8	51·8	55·8	<0·001^e^
Maternal pre-pregnancy BMI, % ≥25kg/m^2^	2,450	23·6	20·0	18·2	14·2	0·001^e^
Maternal education, % up to A-level/Degree	2,595	46·1	49·1	49·2	58·7	<0·001^e^
Highest household social class, % Grade I/II	2,532	28·4	28·8	32·6	42·9	<0·001^e^
Moderate-to-vigorous physical activity at 11 years, % ≥20 mins/day	2,335	54·0	49·8	46·1	48·7	0·175^e^
**C-EWG score at 10 years, n**	**2,739**	**341**	**741**	**784**	**873**	
Age^b^, mean (sd) years	2,739	10·6 (0·2)	10·6 (0·2)	10·6 (0·2)	10·6 (0·2)	0·899^d^
BMI^c^, mean (sd) kg/m^2^	2,720	18·5 (3·2)	18·6 (3·3)	17·8 (2·8)	17·9 (2·9)	0·004^d^
Total Energy, mean (sd) MJ/day	2,739	8·3 (1·5)	8·1 (1·5)	7·8 (1·5)	7·6 (1·6)	<0·001^d^
Sex, % female	2,739	49·9	52·9	53·1	55·0	0·444^e^
Validity of dietary reporting, % Under-reporters	2,731	26·3	26·2	33·5	37·3	<0·001^e^
Maternal age at delivery, % ≥30 years	2,632	45·7	47·0	53·5	54·4	0·003^e^
Maternal pre-pregnancy BMI, % ≥25kg/m^2^	2,450	23·4	17·7	18·2	15·7	0·032^e^
Maternal education, % up to A-level/Degree	2,595	39·6	46·5	53·3	60·8	<0·001^e^
Highest household social class, % Grade I/II	2,532	23·8	29·5	35·7	42·4	<0·001^e^
Moderate-to-vigorous physical activity at 11 years, % ≥20 mins/day	2,335	48·3	45·1	52	49·3	0·095^e^
**C-EWG score at 13 years, n**	**2,739**	**277**	**554**	**756**	**1,152**	
Age^b^, mean (sd) years	2,739	13·8 (0·2)	13·8 (0·2)	13·8 (0·2)	13·8 (0·2)	0·054^d^
BMI^c^, mean (sd) kg/m^2^	2,735	20·4 (3·5)	19·8 (3·1)	20·1 (3·1)	20·3 (3·3)	0·008^d^
Total Energy, mean (sd) MJ/day	2,739	9·1 (1·9)	8·9 (1·9)	8·2 (2·1)	7·7 (2·1)	<0·001^d^
Sex, % female	2,739	38·3	46·2	53·4	60·1	<0·001^e^
Validity of dietary reporting, % Under-reporters	2,724	51·3	46·2	61·9	68·7	<0·001^e^
Maternal age at delivery, % ≥30 years	2,632	47·2	49·4	49·9	53·5	0·098^e^
Maternal pre-pregnancy BMI, % ≥25kg/m^2^	2,450	19·4	20·7	18·3	16·0	0·130^e^
Maternal education, % up to A-level/Degree	2,595	46·6	46·4	50·8	57·0	<0·001^e^
Highest household social class, % Grade I/II	2,532	27·0	32·3	32·6	39·0	<0·001^e^
Moderate-to-vigorous physical activity at 13 years ≥20 mins/day	2,123	57·4	50·9	53·2	47·7	0·031^e^

Abbreviations: C-EWG score; Chilren’s Eatwell Guide Score. ^a^Eatwell Guide score includes sum of score obtained from adherence to (1 point) or non adherence to (0 points) the following nine foods and nutrients: total fat, saturated fat, free sugars, fibre, salt, oily fish, non-oily fish, fruit and vegetables and red and processed meat). ^b^Age refers to age at each point of dietary data collection (7, 10, 13 years). ^c^BMI corresponds to measurements taken at each time point of dietary data collection. ^d^P-value calculated using Kruskal–Wallis test. ^e^P-values calculated using chi-squared test.

Tables 3 and 4 show the results for the unadjusted, minimally adjusted and fully adjusted associations between the C-EWG score (categorical and continuous) at 7, 10 and 13 years and CMR score at 17 years and 24 years, respectively. In fully adjusted models, each 1-point increase in C-EWG at 7 years was associated with a decrease in CMR score at 17 years (*β* −0·13; 95 % CI −0·25, −0·01). When the C-EWG at 7 years was analysed as a categorical variable, the association with the CMR score at 17 years was not apparent. There was no evidence of an association between the C-EWG at 10 and 13 years and CMR score at 17 years. A higher C-EWG score at 7, 10 and 13 years was related to a decrease in CMR score at 24 years; however, at 10 and 13 years, this association was only evident when the C-EWG was analysed as a continuous variable. The strongest association was between the C-EWG score at 7 years and CMR at 24 years (in fully adjusted models *β* −0·96; 95 % CI −1·54, −0·37 for ≥ 3 compared with 0 C-EWG points and *β* −0·25; 95 % CI −0·38, −0·13 for each 1-point increment in C-EWG score). The results from a complete-case analysis (online Supplementary Tables 1 and 2) showed similar findings. The likelihood ratio test supported the assumption that the associations were linear (*P* > 0·1) in all the combinations of exposures and outcomes.

**Table 3.** Multivariable linear regression models for the relationship between the children’s Eatwell Guide (C-EWG) score at 7, 10 and 13 years and cardiometabolic risk score at 17 years, using imputed datasets in the ALSPAC cohort (n=1,940)

Children’s Eatwell Guide (C-EWG) score by participant age^a^	Cardiometabolic Risk (CMR) z-score at 17 years									
		Unadjusted			Minimally Adjusted^b^			Fully Adjusted^c^		
	N	ß (95%CI)		P-value	ß (95%CI)		P-value	ß (95%CI)		P-value
***C-EWG score - 7 years***										
0 points	223	Reference			Reference			Reference		
1 point	508	0·05	(–0·52, 0·61)	0·874	–0·02	(–0·59, 0·54)	0·944	–0·05	(–0·61, 0·48)	0·860
2 points	522	–0·33	(–0·90, 0·23)	0·247	–0·47	(–1·04, 0·09)	0·102	–0·47	(–1·03, 0·10)	0·106
≥3 points	687	–0·41	(–0·96, 0·13)	0·136	–0·59	(–1·13, –0·04)	0·035	–0·51	(–1·06, 0·04)	0·069
Continuous^d^	1,940	–0·16	(–0·28, 0·04)	0·007	–0·16	(–0·28, –0·04)	0·007	–0·13	(–0·25, –0·01)	0·032
***C-EWG score - 10 years***										
0 points	241	Reference			Reference			Reference		
1 point	540	0·24	(–0·31, 0·79)	0·392	0·24	(–0·30, 0·79)	0·374	0·29	(–0·25, 0·83)	0·253
2 points	548	0·05	(–0·49, 0·60)	0·850	0·01	(–0·56, 0·53)	0·961	0·14	(–0·41, 0·68)	0·616
≥3 points	611	–0·20	(–0·74, 0·34)	0·467	–0·29	(–0·82, 0·25)	0·296	–0·10	(–0·64, 0·44)	0·726
Continuous^d^	1,940	–0·09	(–0·21, 0·03)	0·161	–0·12	(–0·24, 0·01)	0·064	–0·06	(–0·18, 0·07)	0·353
***C-EWG score - 13 years***										
0 points	207	Reference			Reference			Reference		
1 point	396	0·34	(–0·26, 0·95)	0·265	0·40	(–0·21, 1·00)	0·197	0·40	(–0·20, 1·00)	0·190
2 points	529	0·30	(–0·28, 0·88)	0·305	0·21	(–0·36, 0·79)	0·466	0·25	(–0·33, 0·82)	0·402
≥3 points	808	0·42	(–0·13, 0·97)	0·131	0·27	(–0·29, 0·82)	0·343	0·36	(–0·20, 0·91)	0·206
Continuous^d^	1,940	0·06	(–0·05, 0·18)	0·274	0·01	(–0·10, 0·12)	0·863	0·04	(–0·07, 0·16)	0·447

Abbreviations: Q-Quartile. ^a^C-Eatwell Guide (C-EWG) score consists of 9 recommended foods and nutrients. ^b^Minimally adjusted regression model adjusted for sex and dietary misreporting. ^c^Fully Adjusted: Multivariable regression model adjusted for sex, dietary misreporting, maternal highest education level, family highest social class and physical activity level at 11 years (for analysis of C-EWG score at 7 and 10 years) and physical activity at 13 years (for analysis of C-EWG score at 13 years). ^d^Estimated mean change in CMR z-score associated with a 1 point increase in C-EWG score.

**Table 4.** Multivariable linear regression models for the relationship between the children’s Eatwell Guide (C-EWG) score at 7, 10 and 13 years and cardiometabolic risk score at 24 years, using imputed datasets in the ALSPAC cohort (n = 1,957)

Children’s Eatwell Guide (C-EWG) score by participant age^a^	Cardiometabolic Risk (CMR) z-score at 24 years									
		Unadjusted			Minimally Adjusted^b^			Fully Adjusted^c^		
	N	ß (95%CI)		P-value	ß (95%CI)		P-value	ß (95%CI)		P-value
***C-EWG score - 7 years***										
0 points	209	Reference			Reference			Reference		
1 point	480	–0·22	(–0·83, 0·40)	0·489	–0·24	(–0·86, 0·37)	0·436	–0·27	(–0·88, 0·34)	0·402
2 points	560	–0·55	(–1·15, 0·05)	0·073	–0·69	(–1·29, –0·09)	0·025	–0·68	(–1·28, –0·08)	0·033
≥3 points	708	–0·92	(–1·51, –0·34)	0·002	–1·10	(–1·68, –0·51)	<0·001	–0·96	(–1·54, –0·37)	0·001
Continuous^d^	1,957	–0·25	(–0·38, –0·13)	0·001	–0·30	(–0·43, –0·85)	<0·001	–0·25	(–0·38, –0·13)	0·001
***C-EWG score - 10 years***										
0 points	242	Reference			Reference			Reference		
1 point	513	0·23	(–0·35, 0·81)	0·441	0·21	(–0·36, 0·79)	0·466	0·26	(–0·32, 0·83)	0·377
2 points	558	–0·25	(–0·82, 0·32)	0·390	–0·32	(–0·89, 0·25)	0·271	–0·14	(–0·71, 0·43)	0·637
≥3 points	644	–0·62	(–1·18, –0·06)	0·029	–0·76	(–1·32, –0·19)	0·009	–0·53	(–1·09, 0·04)	0·066
Continuous^d^	1,957	–0·23	(–0·36, –0·10)	<0·001	–0·27	(–0·40, –0·14)	<0·001	–0·20	(–0·33, –0·08)	0·002
***C-EWG score - 13 years***										
0 points	181	Reference			Reference			Reference		
1 point	398	–0·26	(–0·93, 0·40)	0·439	–0·25	(–0·92, 0·41)	0·456	–0·24	(–0·91, 0·42)	0·471
2 points	533	–0·47	(–1·11, 0·18)	0·155	–0·58	(–1·23, 0·06)	0·077	–0·53	(–1·17, 0·11)	0·105
≥3 points	845	–0·49	(–1·10, 0·12)	0·115	–0·69	(–1·31, –0·07)	0·030	–0·55	(–1·17, 0·07)	0·082
Continuous^d^	1,957	–0·12	(–0·23, 0·01)	0·061	–0·17	(–0·29, –0·05)	0·006	–0·12	(–0·24, 0·00)	0·053

Abbreviations: Q-Quartile. ^a^C-Eatwell Guide (C-EWG) score consists of 9 recommended foods and nutrients. ^b^Minimally adjusted regression model adjusted for sex and dietary misreporting. ^c^Fully Adjusted: Multivariable regression model adjusted for sex, dietary misreporting, maternal highest education level, family highest social class and physical activity level at 11 years (for analysis of C-EWG score at 7 and 10 years) and physical activity at 13 years (for analysis of C-EWG score at 13 years). ^d^Estimated mean change in CMR z-score associated with a 1 point increase in C-EWG score.

Tables 5 and 6 present the association between the C-EWG scores at each age (per 1-point increment) and separate CMR factors (anthropometrics, blood lipids, blood pressure and glucose metabolism) at 17 and 24 years, respectively. An increase in C-EWG score at 7 years was associated with a decrease in several CMR markers at 17 years (BMI, FMI, MAP and systolic blood pressure) and at 24 years (BMI, FMI, waist circumference, LDL-cholesterol, MAP and HOMA-IR). The C-EWG score at 10 years was related to a decrease in FMI z-score at 17 years, and a decrease in BMI, FMI, waist circumference and HOMA-IR at 24 years. An increase in C-EWG score at 13 years was not associated with individual CMR markers at 17 years but was associated with a decrease in anthropometric and glucose metabolism markers at 24 years. In general, the effect sizes were all relatively small.

**Table 5.** Association between the children’s Eatwell Guide (C-EWG) score at 7, 10 and 13 years and individual cardiometabolic risk factors at 17 years, using imputed datasets in the ALSPAC cohort (n = 1,940)

CMR component z-score at 17 years (n = 1,940)	Children’s Eatwell Guide score (C-EWG), per 1-unit increment					
	C-EWG score at 7 years		C-EWG score at 10 years		C-EWG score at 13 years	
	**ß (95%CI)** ^**a**^	P-value	**ß (95%CI)** ^**a**^	P-value	**ß (95%CI)** ^**a**^	P-value
***Anthropometric***						
Body Mass Index	–0·06 (–0·09, –0·02)	0·001	–0·04 (–0·08, 0·01)	0·097	–0·02 (–0·05, 0·02)	0·312
Fat Mass Index^b^	–0·06 (–0·09, –0·03)	<0·001	–0·05 (–0·09, –0·01)	0·018	–0·02 (–0·05, 0·01)	0·258
***Blood lipids***						
Total cholesterol	–0·02 (–0·06, 0·01)	0·217	–0·01 (–0·02, 0·05)	0·487	–0·00 (–0·04, 0·02)	0·63
HDL cholesterol^b^	0·01 (–0·02, 0·05)	0·479	0·01 (–0·03, 0·04)	0·735	0·03 (–0·00, 0·06)	0·052
LDL cholesterol^b^	–0·02 (–0·05, 0·01)	0·252	0·02 (–0·02, 0·05)	0·296	–0·00 (–0·03, 0·03)	0·942
Triacylglycerol^b^	0·01 (–0·02, 0·05)	0·402	–0·01 (–0·02, 0·05)	0·415	0·03 (0·00, 0·06)	0·051
***Blood pressure***						
Systolic BP	–0·05 (–0·09, –0·02)	0·003	–0·03 (–0·06, 0·01)	0·099	–0·00 (–0·03, 0·03)	0·955
Diastolic BP	–0·03 (–0·06, 0·006)	0·110	–0·01 (–0·05, 0·02)	0·425	0·00 (–0·03, 0·03)	0·983
Mean Arterial BP^b^	–0·04 (–0·08, 0·009)	0·013	–0·02 (–0·06, 0·01)	0·189	0·00 (–0·30, 0·03)	0·996
***Glucose metabolism***						
Insulin	–0·03 (–0·06, 0·01)	0·147	–0·02 (–0·06, 0·01)	0·224	–0·00 (–0·04, 0·03)	0·808
Glucose	–0·02 (–0·05, 0·02)	0·397	0·01 (–0·02, 0·05)	0·505	0·01 (–0·02, 0·04)	0·457
HOMA-IR^b^	–0·03 (–0·06, 0·00)	0·077	–0·02 (–0·05, 0·02)	0·282	–0·00 (–0·03, 0·03)	0·882

Abbreviations: C-EWG: childrens Eatwell Guide (C-EWG); CMR score: Cardiometabolic risk score. HOMA-IR: Homeostatic Model Assessment of Insulin Resistance. BP: Blood Pressure. HDL cholesterol: High-density lipoprotein cholesterol. LDL cholesterol: low-density lipoprotein cholesterol. ^a^Beta coefficients (95% confidence intervals) derived from multivariable linear regression models adjusted for sex, dietary misreporting, physical activity at 11 and 13 years, mother’s highest education level, highest family social class. ^b^Cardiometabolic parameters included in the Cardiometabolic Risk Score.

**Table 6.** Association between the children’s Eatwell Guide (C-EWG) score at 7, 10 and 13 years and individual cardiometabolic risk factors at 24 years, using imputed datasets in the ALSPAC cohort (n = 1,957)

CMR components at 24 years (n=1,957), z-scores	Children’s Eatwell Guide score (C-EWG), per 1-unit increment					
	C-EWG score at 7 years		C-EWG score at 10 years		C-EWG score at 13 years	
	**ß (95%CI)** ^**a**^	P-value	**ß (95%CI)** ^**a**^	P-value	**ß (95%CI)** ^**a**^	P-value
***Anthropometric***						
Body Mass Index	–0·07 (–0·10, 0·03)	<0·001	–0·06 (–0·09, –0·03)	<0·001	–0·05 (–0·08, –0·01)	0·005
Fat Mass Index^b^	–0·07 (–0·10, –0·03)	<0·001	–0·07 (–0·10, –0·03)	<0·001	–0·05 (–0·09, –0·02)	0·001
Waist circumference	–0·07 (–0·11, –0·04)	<0·001	–0·80 (–0·11, –0·04)	<0·001	–0·04 (–0·07, –0·00)	0·034
***Blood lipids***						
Total cholesterol	–0·03 (–0·06, 0·01)	0·103	0·00 (–0·03, 0·03)	0·010	–0·01 (–0·04, 0·03)	0·718
HDL cholesterol^b^	–0·02 (–0·05, 0·10)	0·225	–0·03 (–0·07, 0·00)	0·067	–0·01 (–0·05, 0·02)	0·407
LDL cholesterol^b^	–0·04 (–0·07, –0·01)	0·018	0·02 (–0·05, 0·01)	0·360	–0·01 (–0·05, 0·02)	0·418
Triacylglycerol^b^	–0·00 (–0·03, 0·03)	0·984	–0·00 (–0·04, 0·03)	0·806	0·00 (–0·03, 0·04)	0·883
***Blood pressure***						
Systolic BP	–0·07 (–0·10, –0·03)	<0·001	–0·03 (–0·06, 0·01)	0·129	–0·02 (–0·05, 0·01)	0·248
Diastolic BP	–0·05 (–0·08, –0·02)	0·003	–0·02 (–0·05, 0·02)	0·352	–0·02 (–0·05, 0·01)	0·250
Mean Arterial BP^b^	–0·06 (–0·10, –0·03)	<0·001	–0·02 (–0·06, 0·01)	0·192	–0·02 (–0·05, 0·01)	0·198
***Glucose metabolism***						
Insulin	–0·05 (–0·08, –0·02)	0·003	–0·06 (–0·09, –0·02)	0·001	–0·04 (–0·07, –0·00)	0·025
Glucose	–0·00 (–0·04, 0·03)	0·953	–0·01 (–0·05, 0·02)	0·486	0·00 (–0·03, 0·04)	0·831
HOMA-IR^b^	–0·05 (–0·09, –0·02)	0·000	–0·06 (–0·09, –0·02)	0·001	–0·03 (–0·06, 0·00)	0·063

Abbreviations: C-EWG: childrens Eatwell Guide (C-EWG); CMR score: Cardiometabolic risk score. HOMA-IR: Homeostatic Model Assessment of Insulin Resistance. BP: Blood Pressure. HDL cholesterol: High-density lipoprotein cholesterol. LDL cholesterol: low-density lipoprotein cholesterol. ^a^Beta coefficients (95% confidence intervals) derived from multivariable linear regression models adjusted for sex, dietary misreporting, physical activity at 11 and 13 years, mother’s highest education level, highest family social class. ^b^Cardiometabolic parameters included in the Cardiometabolic Risk Score.

## Discussion

The dietary intakes of school-age children in this population-based UK birth cohort were largely suboptimal compared with national dietary recommendations, with over half the children meeting fewer than three of the nine C-EWG recommendations. Meeting more UK dietary recommendations at 7 years of age (indicated by higher C-EWG scores) was related to better overall cardiometabolic health (lower CMR scores) at 17 and 24 years. There was also some evidence, although weaker, that adhering to more dietary recommendations at 10 years resulted in a better CMR profile at 24 years. To our knowledge, this is the first study to assess how overall adherence to UK dietary guidelines in childhood is associated with future cardiometabolic health. Our results add to the UK-based evidence on the health benefits of following government dietary recommendations. Above all, the findings suggest that a better diet quality throughout middle childhood, reflected by greater adherence to dietary guidelines, may have a positive influence on cardiometabolic health in young adulthood. This underscores the importance of establishing healthy dietary habits in line with national dietary recommendations starting from the early years.

Our findings are in line with recent research within the Generation-R study, a population-based cohort study in the Netherlands, which also calculated a diet quality score to reflect adherence to age-specific national dietary guidelines^(49)^. They found that a higher diet quality score at 8 years of age was associated with a lower CMR factor score at 10 years (*β* –0·08 (95 % CI −0·15, –0·001)). A cross-sectional study of 6–8-year-olds within the PANIC Finnish cohort study also observed that higher adherence to a Finnish Children Healthy Eating Index was related to better cardiometabolic health, but only in boys (*β* –0·14 (95 % CI 0·27, 0·00))^(50)^. Our results also align with several cross-sectional studies in adults which reported that greater adherence to dietary guideline indices was inversely related to the metabolic syndrome and metabolic risk factors^(4,9,13,51)^.

The association between the C-EWG and cardiometabolic health at 24 years was stronger when dietary intake was assessed at 7 years than at 10 or 13 years. This could reflect a more sensitive period during childhood when diet quality has a greater impact on cardiometabolic health in early adulthood. It might also reflect greater challenges in accurately assessing habitual dietary intakes in the older participants, due to more changeable diets as parents become less influential on their eating habits outside the home^(52)^ or increased dietary under-reporting, particularly of socially undesirable foods^(53)^. Indeed, in our study there were higher levels of misreporting of dietary intake at 13 years than at 7 or 10 years. Plausibility of dietary reporting was therefore adjusted for in multivariable regression models. We also observed stronger associations between the C-EWG score and cardiometabolic health at 24 years than at 17 years. This could be because the participants at 17 years were generally cardiometabolically ‘healthier’, reflected by lower mean values and less variation in the individual CMR measurements, than at 24 years. Consequently, our study might have had less power to detect associations at the earlier age group.

Meeting more UK dietary recommendations at 7 years was related to a small beneficial effect on most of the individual cardiometabolic markers at 17 and 24 years (FMI, HOMA-IR, MAP and LDL-cholesterol at 24 but not at 17 years). The C-EWG-CMR score associations at other ages were mainly driven by FMI and HOMA-IR. In contrast, the Generation-R study found that a higher diet quality score was only associated with lower blood pressure, with no significant associations for body fat mass, insulin, TAG or HDL-cholesterol^(49)^ but these were assessed at the very young age of 10 years. In the PANIC study, the children were 6–8 years of age and the only individual CMR factor associated with the Finnish Children Healthy Eating Index was TAG^(50)^. The differences among studies in the construction of the diet recommendations scores, such as which food/nutrients were included, the recommended intakes or constraints, as well as the age of the participants when the CMR factors were assessed, mean that findings between studies cannot be directly compared.

Few other studies have explored how dietary quality indices relate to individual CMR factors in children. A systematic review in 2020 identified 128 *a priori* diet quality indices uniquely designed for and/or used in children and adolescents, but only ten of these indices were analysed in relation to cardiometabolic markers (mainly body composition, lipid profiles and blood pressure)^(54)^. These studies used international diet quality indices or indices reflecting adherence to national dietary guidelines from countries such as Australia, the USA, Belgium and Japan. Approximately half of these studies found that the diet quality index was related to measures of adiposity, which was a consistent finding in our study^(54)^. This is of particular public health relevance, because in the UK 28 % of 4–5-year-olds and 41 % of 10–11-year-olds are overweight or obese (data from the 2020/2021 school year)^(55)^. Of additional concern is the fact that obesity rates in primary school children have recently suffered the highest annual rise out of the last 15 years (4·5 % increase between 2019–2020 and 2020–2021). Moreover, children who are overweight and obese are more likely to be obese in adulthood and are at increased risk of developing chronic disease such as CVD, T2D, the metabolic syndrome and certain cancers^(56,57)^.

Our results related to individual CMR markers are also supported by research within the UK Biobank which reported that adults meeting more WHO dietary recommendations (for saturated fats, free sugars, fibre, and fruit and vegetables) had lower body fat, waist circumference, LDL-cholesterol and TAG. Another study in UK adults found that greater alignment to eight core UK dietary recommendations, assessed using a dietary reference value index, was associated with a decrease in several CMR factors, including waist circumference, BMI, total cholesterol and blood glucose^(7)^.

Our research has several strengths and limitations. Although the C-EWG score is a useful tool to measure overall adherence to key UK dietary recommendations, it does not incorporate all core UKHSA dietary recommendations^(58)^ (i.e. carbohydrates and protein are not included) because we based the score on previously published methodology^(11)^. Another consideration is that several EWG recommendations were different or not in place when the dietary data were collected. For example, the limit for percentage of energy from free sugars was ≤ 10 % at the time of data collection, and it was subsequently decreased to ≤ 5 % in 2015^(34)^. The 5-a-day fruit and vegetables recommendation was launched in March 2003, so would not have been in place when the dietary data were collected for the 7–10-year-olds. However, since our objective was to assess whether adherence to current guidelines was related to cardiometabolic health, this should not affect the internal validity of the observed findings. Another consideration is that each food and nutrient contributed an equal weight towards the total C-EWG score, with no ranking of importance for cardiometabolic health. Although it is likely that there are differences in effects for each food/nutrient, due to the novelty of such diet quality indices there is no guidance on how weighting could be assigned.

Dietary assessment was done via dietary diaries, which can be subject to reporting error and recall bias. The discretionary use of salt (salt added at the table or whilst cooking) can be difficult to accurately quantify using self-reported assessment tools^(59)^. It is therefore possible that salt intake was underestimated in this study and that adherence to salt guidelines was in fact lower^(60)^. However, dietary diaries are normally less susceptible to misreporting than FFQ^(61)^ and we controlled for plausibility of dietary reporting in all regression analyses. A further limitation is that the intake of less frequently eaten foods, such as fish and seafood, may have been underestimated if they were eaten on days not recorded by the 3-d diet diaries, or overestimated if the diet diary days coincided with an infrequent eating occasion. This could result in some children being misclassified into meeting or not meeting the recommendations. Nonetheless, in our study the mean fish intake in the 7–10-year-olds was 6·3 g/4184 kJ/d which was relatively similar to the mean intake of 7·2 g/4184 kJ/d in the nationally representative sample of 6–11-year-olds from the UK 2008–2012 National Diet and Nutrition Survey, which recorded diet over more days (4-d diet diaries)^(62)^.

Although we were unable to adjust for potential confounding by sedentary behaviour, as this information was not available at all three ages, we did adjust for other lifestyle and socio-demographic variables identified as confounders of the C-EWG score–CMR score association. However, residual confounding due to measurement error in these data or other unknown confounding factors, not included, cannot be ruled out.

A further consideration is the generalisability of our results to the UK population, as there was evidence of bias due to sample attrition over the follow-up period (maximum follow-up of 17 years). Although we used multiple imputation of missing data to minimise attrition bias due to incomplete covariate data, and to prevent further reductions in sample size and study power^(63)^, the sub-population of the cohort included in our final analysis was more likely to have a higher socio-economic status and a better cardiometabolic profile. In addition, prior research in ALSPAC children observed that healthier dietary patterns were linked to higher socio-economic status^(64)^. Therefore, the participants included in the present analysis most likely had more homogeneous and healthier dietary patterns than the general population, leading to a potential underestimation of the strength of the association.

A strength of this research is the longitudinal study design which provides a clear distinction between periods of exposure and risk. We also had repeated measures of both exposure (C-EWG score) and outcome (CMR score), so we were able to assess if adherence to dietary guidelines at varying periods throughout childhood had a differential impact on cardiometabolic health. A further advantage is the use of CMR scores, which are particularly valuable when studying cardiometabolic health in adolescence/young adulthood because the variation in individual CMR risk factors may still be too subtle to indicate risk on their own. However, assessing a cluster of CMR measures can provide a relevant pre-clinical outcome when studying diet–disease associations. In addition, continuous CMR scores in children have been shown to be better predictors of CVD in young adults compared with dichotomous measures^(19)^. Prospective studies with longer follow-up times could explore whether meeting national dietary guidelines in childhood predicts incidence of CVD, T2D and other non-communicable chronic diseases later in life.

In this UK population-based cohort, we observed generally very poor adherence to national dietary guidelines; most of the 7–13-year-old children did not consume enough fruit and vegetables, fibre or fish, and in contrast the majority consumed too much fat, particularly saturated fat, and far too much sugar and salt. These suboptimal dietary habits in children are confirmed by more recent UK data from the National Diet and Nutrition Survey showing that in general dietary intakes of UK children and adolescents are still falling short of government dietary targets for many core foods and nutrients^(65,66)^. Therefore, our findings suggest an opportunity for preventative strategies to increase cardiometabolic health in early adulthood by improving diet quality during childhood in line with national dietary guidelines. Establishing healthy dietary habits early in life is crucial, since this is when dietary habits are acquired, and can become embedded and track into adulthood^(64,67)^.

Successfully moving a population’s diet towards recommended dietary targets could also result in wider health benefits: it is estimated that approximately 33 000 deaths/year could be delayed in the UK if the government dietary recommendations for consumption of fruits and vegetables, fibre, salt and dietary fats were met^(12)^. Over 15 000 of the increased years of life would be due to increased consumption of fruit and vegetables^(12)^. However, significant transformations to the entire food environment, involving more dietary-related government policies (akin to the soft drinks industry levy), continued work with the food industry (in line with product specific salt reduction targets) and multi-level strategies to help promote healthier eating habits, would probably be needed to drive sufficient positive dietary changes at a population level. Primordial prevention strategies could also use targeted evidence-based lifestyle interventions (involving diet and physical activity) in children/adolescents with early markers of abnormal cardiometabolic phenotypes.

In conclusion, our findings showed that meeting a greater number of UK dietary guidelines during mid-childhood was related to better overall cardiometabolic health in early adulthood (24 years). This supports the role that dietary quality early in life plays in the life-course development of cardiometabolic alternations, which are risk factors for CVD and T2D. The fact that dietary habits of children in this cohort were generally falling substantially short of dietary recommendations highlights the need for public health initiatives to help improve the quality of UK children’s diets in order to reduce the burden of cardiometabolic diseases later in life.

## Supplementary material

For supplementary material accompanying this paper visit https://doi.org/10.1017/S0007114523000685. click here to view supplementary material
