# Supplementary material for: Prospective association between adherence to UK dietary guidelines in school-age children and cardiometabolic risk markers in adolescence/early adulthood in the Avon Longitudinal Study of Parents and Children (ALSPAC) cohort
Source: Br J Nutr. 2023 Apr 17;130(10):1766–78. doi: 10.1017/S0007114523000685 (PMC10587371; doi:10.1017/S0007114523000685)
Supplement: Supplementary file 1 [file S0007114523000685sup001.docx]

**Supplementary Material: British Journal of Nutrition**

**Prospective association between adherence to UK dietary guidelines in school-aged children and cardiometabolic risk markers in adolescents/early adulthood from the Avon Longitudinal Study of Parents and Children (ALSPAC) cohort.**

Genevieve Buckland^1^, Caroline M Taylor^1^, Pauline M Emmett^1^, Kate Northstone^2^

**^1^Centre for Academic Child Health, Bristol Medical School, University of Bristol**

**^2^**Department of Population Health Sciences**, Bristol Medical School, University of Bristol**

**Supplementary Table 1.** Multivariable linear regression models for the relationship between the children’s Eatwell Guide (C-EWG) score at 7, 10 and 13 years and cardiometabolic risk score at 17 years in the ALSPAC cohort for complete-case analysis (n=1,261).

**Supplementary Table 2.** Multivariable linear regression models for the relationship between the children’s Eatwell Guide (C-EWG) score at 7, 10 and 13 years and cardiometabolic risk score at 24 years, in the ALSPAC cohort for complete-case analysis (n=1,266).

**Supplementary Table 3.** Comparison of characteristics of ALSPAC participants with imputed and observed data.

**Supplementary Table 4.** Intake of key nutrients within the Eatwell Guide in the ALSPAC children at 7, 10 and 13 years, according to adherence to the UK dietary guidelines.

**Supplementary Table 5**. Intake of key foods within the Eatwell Guide in the ALSPAC children at 7, 10 and 13 years, according to adherence to the UK dietary guidelines.

**Supplementary Table 6.** Comparison of baseline characteristics and cardiometabolic risk factors at 17 years and 24 years in ALSPAC participants who had complete dietary and at all three ages (7, 10 and 13 years) and outcome data at 17 years and/or 24 years compared to those with incomplete data on these variables.
